# Efficacy of Hypnosis-Based Treatment in Amyotrophic Lateral Sclerosis: A Pilot Study

**DOI:** 10.3389/fpsyg.2012.00465

**Published:** 2012-11-05

**Authors:** Arianna Palmieri, Johann Roland Kleinbub, Vincenzo Calvo, Gianni Sorarù, Irene Grasso, Irene Messina, Marco Sambin

**Affiliations:** ^1^Department of Philosophy, Sociology, Pedagogy and Applied Psychology, University of PadovaPadova, Italy; ^2^Department of Neurosciences, University of PadovaPadova, Italy

**Keywords:** amyotrophic lateral sclerosis, hypnotherapy, self-hypnosis, psychological intervention

## Abstract

**Background:** Amyotrophic lateral sclerosis (ALS) and its devastating neurodegenerative consequences have an inevitably psychological impact on patients and their caregivers: however, although it would be strongly needed, there is a lack of research on the efficacy of psychological intervention. Our aim was to investigate the effect of hypnosis-based intervention on psychological and perceived physical wellbeing in patients and the indirect effect on caregivers. **Methods:** We recruited eight ALS volunteers patients as a pilot sample for an hypnosis intervention and self-hypnosis training protocol lasting 1 month. Anxiety and depression level was measured in patients and caregivers at pre and post treatment phase. Quality of life and perceived physical symptoms changes were also investigated in patients. **Results:** One month pre-post treatment improvement in depression, anxiety, and quality of life was clearly clinically observed and confirmed by psychometric analyses on questionnaire data. Moreover, decreases in physical symptoms such as pain, sleep disorders, emotional lability, and fasciculations were reported by our patients. Improvements in caregiver psychological wellbeing, likely as a consequence of patients psychological and perceived physical symptomatology improvement, were also observed. **Conclusion:** To the best of our knowledge, even if at a preliminary level, this is the first report on efficacy psychological intervention protocol on ALS patients. The findings provide initial support for using hypnosis and self-hypnosis training to manage some ALS physical consequences and mainly to cope its dramatic psychological implications for patients and, indirectly, for their caregivers.

## Introduction

Amyotrophic Lateral Sclerosis (ALS) is classically characterized by progressive loss of upper and lower motor neurons, causing weakness and hypotrophy of upper and lower limbs, dysphagia and dysarthria, and respiratory failure, which is the most common cause of death. Frequently, patients require intubation and mechanical ventilation and, in a large proportion, receive tracheostomy, leading to inevitably, overwhelming locked-in syndrome (Vianello et al., 2011). Secondary symptoms that affects patient quality of life are also pain (Pagnini et al., 2011a; Chiò et al., 2012; Pagnini, 2012), sleep disorders (Blackhall, 2012), emotional lability (Palmieri et al., 2009), and fasciculations (Rana et al., 2009; De Carvalho and Swash, 2012). Although pharmacology and physiotherapy research efforts, effective treatments for ALS have remained elusive (Beghi et al., 2011). Not surprisingly, a very active community of researchers is dedicated to deepen the devastating psychological impact of ALS in patients and caregivers. Depression and anxiety are two reactions typically described in response of the disease, mainly in the first stage, in which are accompanied by anger, hopelessness, and suicidal ideation (Palmieri et al., 2010a; Pagnini, 2012). Patient psychic status has been found directly related to caregiver’s one (Palmieri et al., 2009; Pagnini et al., 2010a, 2011b). Analogously, caregiver psychological status has a deep impact on patient (Chiò et al., 2005). Although psychological wellbeing is crucial also in determining a better prognosis in ALS (McDonald et al., 1994), there is an absence of research on efficacy of psychological intervention in ALS. That is what was recently highlighted by Pagnini et al. (2012), in a relevant short communication published in ALS leading journal, eloquently entitled: “ALS: time for research on psychological intervention?” The communication arises the importance to carry out exploratory investigation on the impact and the suitability of psychological treatments for ALS patients. Given the devastating physical involvement of the disease, which affects also speech ability and eventually may result in the locked-in syndrome, a mind-body treatment technique, such as hypnosis, mindfulness meditation, or biofeedback (techniques included among complementary therapies in neurology; Wahbeh et al., 2008), could represent the eligible psychological forms of intervention. In particular, probably no other contemporary therapeutic intervention has a longer and corroborated clinical/scientific history than that of hypnosis. Our purpose was to explore, in the form of a pilot study, the applicability and efficacy of a hypnosis-based treatment in a small sample of ALS patients. Since the potential treatment benefit obtained from individual active participation (Bandura, 1997), a self-hypnosis training, other than ericksonian hypnosis classical intervention, was proposed to patients. Our primary hypothesis was that the treatment and the training may determine relevant reductions in the two primary psychological reactions to the disease, i.e., anxiety and depression, and, in general, in quality of life. In addition, we aimed to explore if the participation to the hypnosis training could produce significant improvements in the perception of the above-mentioned physical secondary symptomatology (i.e., pain, sleep disorders, emotional lability, and fasciculations). Finally, we hypothesized an eventual reduction of caregivers’ anxiety and depression, as indirect consequence of patients psychological improvement.

## Materials and Methods

### Participants and procedure

Eight consecutive patients with a diagnosis of probable or definite ALS (in accordance with the Revised El Escorial Criteria; Brooks et al., 2000) presenting to the Motor Neuron Disease Center of Padova University Hospital and their respective caregivers (seven spouses, one daughter) were recruited as volunteers for the pilot study. Patients underwent complete neurological and neuropsychological examination within a week before the first treatment session. Our sample was composed by four males and four females, with mean age of 55 years (SD = 7.14); ALSFRS-r (Cedarbaum et al., 1999) mean score was 35 (SD = 7.1), ranging from 21 to 42; four had bulbar onset, and one of them was completely enable to speak; two were completely enable to move upper limbs. Patient neuropsychological profiles did not revealed any alteration if compared to normative data. Further demographic and clinical characteristics of participants are reported in Table 1. The intervention protocol consisted of four weekly domiciliary sessions of hypnosis-based intervention, administered by a psychologist extensively trained in ericksonian hypnotic method and analgesic hypnosis. All sessions were based on a standardized, general induction followed by individual suggestion modified for the patient according to his/her psychological status and physical symptoms. Hypnotic suggestions were constituted by guided visual imagery oriented with an emphasis on developing physical symptomatology controls, such as muscle pain or emotional lability, sense of resilience, self-consciousness, and illness acceptance. After the hypnosis session, which lasted about 45 min, patient was supported by the aiding of the hypnotherapist to try again in recreating hypnotic status with self-hypnosis technique. The general induction, common for all treatment phases and for all patients, was recorded in a CD audio and was left to each patient who was encouraged to practice at least one time every day. Such procedure is inspired by Jensen et al. (2009, 2011) hypnosis-based protocol, successfully applied on multiple sclerosis patients. Sessions included also a brief clinical interview with patient and/or caregiver (separately) collected by a psychotherapist with 10-years experience with ALS. The interview aimed to investigate the subjective perception of the hypnosis treatment efficacy both on psychological and physical perspective, the frequency of application and the eventual difficulties. Each entirely domiciliary session lasted about 2 h. Immediately before the first and after the last treatment, patients and caregivers psychological variables were collected by means of standardized, Italian-validated *ad hoc* questionnaires, as described in the next paragraph. Patient impressions on physical symptomatology were also systematically recorded. The study protocol was approved by the Ethical Committee of the University of Padova and carried out in accordance with the principles of the Declaration Helsinki as revised in 1983. All the participants signed a consent statement after being informed about the study’s purpose and methods.

**Table 1:** **Participants demographic and clinical characteristics**.

Patient	Age	Gender	Time since diagnosis (months)	Onset	ALSFRS-r	FVC (%)	Caregivers age (years); gender
I	64	*F*	24	Bulbar	42	82	(32); F*
II	55	*M*	15	Limb	38	82	(50); *F*
III	59	*F*	10	Limb	40	86	(57); *M*
IV	54	*M*	26	Limb	21	56	(50); *F*
V	43	*M*	18	Limb	39	92	(40); *F*
VI	53	*M*	40	Limb	30	75	(53); *F*
VII	55	*F*	24	Bulbar	39	91	(56); *M*
VIII	66	*F*	10	Limb	31	72	(69); *M*

*ALS-FRS-r, amyotrophic lateral sclerosis functional rating scale-revised; FVC, forced vital capacity*.

*Daughter; in the other cases, caregiver was the spouse.

### Measures

The Hospital Anxiety and Depression Scale (HADS; Zigmond and Snaith, 1983), a measure largely used to identify caseness of anxiety disorders and depression among non-psychiatric patients (Bjelland et al., 2002), easy to administer and well accepted, was employed for patients and their caregivers. It is divided into an Anxiety subscale (HADS-A) and a Depression subscale (HADS-D) both containing seven intermingled items on a 4-point Likert scale (ranging 0–3).

Patients also underwent the Amyotrophic Lateral Sclerosis Specific Quality of Life – revised (ALSSQOL-r; Simmons et al., 2006; Pagnini et al., 2010b), a 46-items on a 11-point Likert scale (ranging 0–10) derived from interviews with ALS patients. The ALSSQOL-r is composed by six subscale including negative emotion (13 items), interaction with people and their environment (11 items), intimacy (seven items), religiousness and spirituality (four items), physical symptoms (six items), and bulbar functioning (five-items).

The five-items ALS Assessment Questionnaire (ALSAQ-5), a further, brief specific quality of life measure derived from the broader ALSAQ-40 (Jenkinson and Fitzpatrick, 2001; Palmieri et al., 2010b), was also administered to patients. Items are composed on a five-point Likert scale (ranging 1–5). Such measure was introduced, in addition to the more exhaustive, even if different in content, ALSSQOL-r, in the perspective of eventual 3 and 6 months follow-up, in order to obviate eventual drop out in quality of life measurement (such phenomenon could be due to progressive physical impairment that can frustrate responding questionnaire with many items).

Global satisfaction with hypnotic-based intervention score in terms of perceived efficacy was assessed asking patients to rate, after treatment, how satisfied they were with the treatment they received on a 1–5 Likert scale (from one as totally unsatisfied to five as extremely satisfied). Subjective perception of secondary symptoms affecting patient everyday life that could receive benefit from hypnosis-based treatment, namely pain, sleep disorders, emotional lability, and fasciculations, were qualitatively investigated before and after the treatment (for an example, in the time lapse immediately preceding the beginning of the treatment were asked to the patients: “Did you suffer physical pain (e.g., cramps)?” And, if yes, after the treatment was asked them: “Did you notice any improvement during the training period?”). To not overfatigue the patients with wide many items standardized measurements, we opted, at this preliminary level, to investigate such physical perceived changes with a simple open-answers interview, avoiding dimension-specific questionnaires for peculiar physical dysfunctions. Due to severity of motor impairment, not every patient would be able to fulfill self-report questionnaires responses. Therefore, to obviate eventual heterogeneity in the data collection, a trained psychologist administered all questionnaires in an eterodirected way.

### Data analysis

Pre-post differences in scores for each subscale of the questionnaires (HADS, ALSSQOL, ALSAQ-5) were analyzed for significance through Wilcoxon Signed Rank Tests at the 0.05 level, and the Cliff’s delta (∂) statistic was used to assess effect size. Cliff’s delta (Cliff, 1993) was chosen as a more robust alternative to the common Cohen’s *d* in conditions were non-parametric statistics are advisable, such as small sample sizes where it is not safe to assume a normal distribution (Hess and Kromrey, 2004). An high effect size is considered ∂ ≥ |0,474|, a medium effect size is ∂ = |0,33| and a low effect size is ∂ < |0,147| (Cohen, 1988). Analysis were performed with R software, version 2.15.1.

## Results

Patients declared to have successfully practiced self-hypnosis at least once every day in the great majority of the days during month treatment. The HADS scores showed that before the treatment two out of eight patients suffered from “mild” depression while the remaining six fell in the normality range; after the treatment all eight patients fell in the normality range for depression. Before the treatment, three patients out of eight suffered from “mild” anxiety, one suffered from “severe” anxiety, and the remaining four patients fell into the normality range. After the treatment, two out of eight patients suffered from “mild” anxiety while the six remaining had a normal score (of note, one was previously ranged as “moderate” and one was previously ranged as “severe”). Pre and post analysis of the HADS scores showed significant reduction in the anxiety subscale, with very high effect sizes (where an high effect size is considered ∂ ≥ |0,474|) both for patients (*W* = 36, *p* = 0.008; ∂ = −1) and caregivers (*W* = 21, *p* = 0.031; ∂ = −0.75); moreover, the HADS depression subscale showed a significant reduction in score with high effect size (*W* = 28, *p* = 0.016; ∂ = −0.875) for the patients group only. Significant pre-post differences were also observed in the ALSSQOL-r average total score (*W* = 1, *p* = 0.016; ∂ = 0.75), negative emotion subscale (*W* = 0, *p* = 0.008; ∂ = 1), and religiosity/spirituality subscale (*W* = 1, *p* = 0.031; ∂ = 0.625). The interaction (*W* = 5, *p* = 0.078; ∂ = 0.75) and physical symptoms (*W* = 0, *p* = 0.125; ∂ = 0.75) subscales’ pre-post differences were not found significant, but still showed a large effect size, whereas the bulbar and intimacy subscales showed only a medium to small effect size. ALSAQ-5 questionnaire did not show any significant difference before and after the treatment (*W* = 16, *p* = 0.219; ∂ = 0.25). Patient and caregiver questionnaires mean and standard deviation score, and pre to post treatment comparison in terms of statistical indices are shown in Table 2.

**Table 2:** **Patient pre and post treatment psychological measures**.

Patient outcome variables (questionnaire scores)	Pre treatment mean (SD)	Post treatment mean (SD)	*P* (Wilcoxon W index)	Pre-post effect size (Cliff’s delta)
HADS anxiety	8.12 (4.58)	4.5 (4.44)	0.008** (36)	−1
HADS depression	4.87 (2)	3 (2.33)	0.016* (28)	−0.87
ALSSAQOL-r negative emotions	7.38 (1.21)	8.44 (0.60)	0.008** (0)	−1
ALSSAQOL-r interaction	7.49 (1.21)	8.21 (1.17)	0.078 (5)	0.75
ALSSAQOL-r intimacy	6.21 (2.17)	6.28 (2.25)	0.437 (6.5)	0.25
ALSSAQOL-r religiousness	8.10 (2.34)	7.12 (1.76)	0.031* (1)	0.62
ALSSAQOL-r physical symptoms	7.06 (1.96)	7.71 (1.53)	0.125 (0)	0.75
ALSSAQOL-r bulbar functioning	6.82 (0.96)	6.95 (1.01)	0.250 (2)	0.37
ALSSAQOL-r total score	6.95 (0.99)	7.68 (0.89)	0.016* (1)	0.75
ALSAQ-5	45 (17.52)	41.87 (20.86)	0.219 (16)	0.25
**CAREGIVER OUTCOME VARIABLES (QUESTIONNAIRE SCORES)**				
HADS anxiety	8 (4.66)	6.12 (3.88)	0.031* (21)	−0.75
HADS depression	4.62 (4.78)	3.25 (5.33)	0.22 (22)	−0.37

*HADS, hospital anxiety and depression scale; ALSSQOL-r, amyotrophic lateral sclerosis specific quality of life – revised; ALSAQ-5, 5-items amyotrophic lateral sclerosis assessment questionnaire*.

Significant results are in bold; *p < 0.05; **p < 0.01.

Regarding physical symptoms, both the two patients who were suffering from sleep disorders, four out of seven patients suffering from muscular pain and cramps, four out of five patients who were suffering fasciculations and each of the four patients suffering from emotional lability declared that their symptoms improved during the training period. A question on the perceived usefulness of the training, ranging from 0: “not useful at all” to 5: “very useful,” showed an average score of 4.25 (SD = 0.71).

## Discussion

In contrast with the prolific scientific literature addressed to investigate the psychological impact of ALS on affected patients (Felgoise et al., 2010; Palmieri et al., 2010a; Lulé et al., 2012; Pagnini, 2012), the need to outline research on efficacy of psychological intervention has recently emerged (Pagnini et al., 2012). The current pilot study represents a preliminary response to such a relevant issue. Our psychological treatment, on a small patient sample size, was focused on hypnosis-based domiciliary intervention (hypnosis intervention and self-hypnosis training) and was designed to address three related goals: to assess the effect of such intervention on both reducing psychological suffering in ALS patients and giving relief from physical symptoms of the disease and consequently contributing to a positive psychological impact on caregivers. Our findings provided a clearly encouraging, although initial, support for using hypnosis to manage psychological and some physical consequences of ALS. In particular, results showed ALS patient improvements in depressive, anxiety symptomatology, and in general quality of life after 1 month treatment. Moreover, relevant pre to post treatment improvements in pain, disorders, emotional lability, and fasciculations were reported and attributed by patients to hypnosis-based intervention and self-hypnosis training. A satisfaction level from good to extreme was finally reported by patients involved in our study, confirming the appreciation and the applicability of the treatment. Caregiver depression and anxiety levels were observed to decrease during 1 month patient treatment, and such positive trend was attributed by them to patient gained psychological wellbeing.

Before the beginning of the treatment, two of our patients were classified as affected by “mild” depression and half of them as affected by “mild” to “severe” level of anxiety. Moreover, at the end of the treatment, the great majority of patients qualitatively showed, from a clinical point of view, a clear amelioration in such aspects, and the global psychometric analyses, calculated on HADS questionnaire scores, confirmed such clinical evidence. These findings on patient psychopathological remission, even if should be considered with caution at this preliminary stage of investigation, are undoubtedly of great interest for clinicians. As an example, depression and anxiety appear to be strongly related in the disease: Atassi et al. (2011) found that anxiety level resulted the unique ALS related symptom that can predict the occurrence of depression. Prevalence of depression in ALS has been reported as highly variable, depending by specific interview methods and assessment tools of investigation (Taylor et al., 2010), and could reach levels as high as 75% (Wicks et al., 2007). The nature of the wide difference in estimation of mood states in ALS is conceivably due also by the fact that the existential despair some patient feel in the face of their illness may be poorly described by the word of “depression.” Their experience of overwhelming sense of demoralization, hopelessness, anger, and loss of meaning in the face of their mortality is something different of feel just depressed (Blackhall, 2012).

Our study highlighted also positive psychological changes in patients’ quality of life after the training. Both specific ALS measures of the quality of life employed in the study, namely the ALSSAQOL (Simmons et al., 2006) and the ALSAQ-5 (Jenkinson and Fitzpatrick, 2001; Palmieri et al., 2010b), showed a clear improvement when comparing pre to post treatment mean scores. In particular, the ALSSAQOL “negative emotion” and “spiritually/religiousness” subscales reached the psychometric significance in terms of pre to post treatment improvements, while “interaction” and “physical symptoms” subscales appeared to be marginally significant. In the palliative care field, improving or maintain global quality of life is considered the crucial challenge in patients whose disease is not responsive to curative treatment as ALS. World Health Organization (WHO) scientific panel clearly established that the main objective of palliative care is achievement of the best quality of life for patients and their families until death (Doyle et al., 2003).

Interestingly, qualitative positive results have been emerged also in the disease secondary symptomatology, as it is perceived by patients. All of them declared to have perceived an improvement, from mild to extreme depending on cases, in pain, sleep disorders emotional lability, or fasciculation syndrome, whereas there was an occurrence before treatment. Since ALS devastating impact on motor system and dysphagic/dysartric, respiratory dysfunctioning, the impression is that secondary symptoms patient suffering have been underestimated by scientific attentional focus. However, recent clinical/epidemiological investigation highlighted such relevant disease consequences for patients. In particular, Chiò et al. (2012), in a wide epidemiological study, show that pain (e.g., muscle spasms, contractures, spasticity, abnormal stresses on the musculoskeletal system imposed by weak musculature) is frequent in all stages of ALS, but it often goes underrecognized and undertreated, recommending research efforts toward the appropriate ALS pain treatment. Our intervention protocol could be interpreted as addressed in such direction as well. The arena in which the hypnosis has probably proved its efficacy most adequately is, indeed, that of hypnotically induced analgesia, providing reduction of both chronic (e.g., cancer) and acute (e.g., painful medical procedure) pain (Freeman et al., 1999). As for ALS sleep disorders (resulting from factors such as reduced mobility, muscle cramps and anxiety, restless legs, and increased myoclonic activity), it may produce daytime symptoms and impairment activities of daily living and can be further affected by an increased incidence of depression (Hetta and Jansson, 1997). In emotional lability and fasciculation syndrome, respectively underestimated source of deep embarrassment (Palmieri et al., 2009) and physical discomfort (Rana et al., 2009), as well for pain and sleep disorders, seems to play a psychological etiopathogenetic non-neglectable role (Palmieri et al., 2009; Rana et al., 2009). As a final confirmation of the treatment approval, in the overall judgment of satisfaction we asked to the patients, all of them declared a good from extreme satisfaction.

Relevant improvement in terms of depression and anxiety reduction, measured with the same patient assessment tools, was reported by caregivers. When asked, the great majority of them stated they were satisfied of hypnotic treatment and self-hypnosis training patient positive outcome, and that the improvement on patient psychological functioning, according to them, has lead to a probable effect on their own mood tone as well. The impact of patient suffering on family caregivers is a further understudied but important topic. Associations of patient distress with caregiver negative affect has recently been shown by some authors (Chiò et al., 2005; Gauthier et al., 2007; Boerner and Mock, 2012). On the other hand, patient’s loss of physical functions was positively related with caregiver burden, anxiety, and somatic expression of depression (Pagnini et al., 2010a).

Our findings on treatment efficacy, in their globality, could appear surprising, but it should be mentioned the number of Authors who have recently enhanced the potentiality of hypnosis-based treatment, and, in general, mind-body techniques, leading to a rapid improvement on anxiety, and depression especially when they are reactive to a medical condition (Shih et al., 2009; Willemsen et al., 2010; Plaskota et al., 2012). Specifically, in the realm of neurological disorders, hypnosis has been reported as an effective adjunctive treatment for organic brain damage (Sullivan et al., 1974), Parkinson disease (Wain et al., 1990), stroke (Holroyd and Hill, 1989), peripheral nerve lesions (Pajntar et al., 1980), cases of organic paralysis (Lucas et al., 1981), various type of dystonia (De Benedittis, 1996), multiple sclerosis (Jensen et al., 2009, 2011), migraine (Ezra et al., 2012), and fibromialgy (Bernardy et al., 2011).

Although our results show promising and exciting insights, the study suffers of a number of limitations, including the typical ones that naturally characterize pilot studies. Primary among them, the small sample size have limited our ability to detect whole treatment effect from a psychometric point of view. A further criticism is the lack of a different treatment condition (e.g., a different mind-body intervention or psychopharmacological treatment) that controls for the effects of time, placebo condition, and expectancy effect. Lastly, additional measurement at pre treatment level in order to investigate psychological traits such as personality profile, attachment style and hypnotic absorption, dissociation, and suggestion ability could have been undoubtedly important variables to be compared with the treatment efficacy (Primavera and Patterson, 1991). Moreover it will be relevant to consider the frequency of self-hypnosis and verify its role in the efficacy of this kind of treatment with ALS patients.

In conclusion, our general aim was to lay some foundations to develop, in a future perspective, a protocol of eligible psychological interventions for ALS, “useful for a meaningful improvement in quality of life or reduction in psychological distress in patients and their caregivers” (Pagnini et al., 2012) and suitable to be proposed to patients with a such peculiar condition, involving bulbar dysfunctions and that can eventually lead to a locked-in syndrome. Despite the pilot study limitations, the findings provide clear support for the potential efficacy of hypnosis-based intervention to cope with devastating consequences of ALS. Further longitudinal researches using larger sample to help determine the reliability of the current findings are warranted.

## Conflict of Interest Statement

The authors declare that the research was conducted in the absence of any commercial or financial relationships that could be construed as a potential conflict of interest.
